# Characterization and Expression Analysis of Sugar Transporters through Partial Least Square Structural Equation Model (PLS-SEM) Revealed Their Role in Pepper (*Capsicum annuum* L.)

**DOI:** 10.3390/plants13131825

**Published:** 2024-07-03

**Authors:** Pan Xia, Shiyong Zhou, Xiaoxue Zhao, Changling Zhao

**Affiliations:** 1 Faculty of Agronomy and Biotechnology, Yunnan Agricultural University, Kunming 650201, China; 2 Faculty of Animal Science and Technology, Yunnan Agricultural University, Kunming 650201, China

**Keywords:** sugar transporter, fruit development, stress treatment, PLS-SEM

## Abstract

Pepper (*Capsicum annuum* L.) is one of the most important economic crops in the world. By controlling the transport and distribution of photosynthetic products between cells and organs, sugar transporters are widely involved in growth and development, environmental adaptation, and microbial interactions. The present study was carried out at the genome-wide level to systematically characterize sugar transporters. As a result, 50 *MST*, 3 *SUT*, and 29 *SWEET* genes were identified and classified. The expression pattern of sugar transporters in pepper was analyzed by transcriptomic data. The expression properties of sugar transporters were further explored in pepper varieties with significant differences in weight, shape, and pungency. It was shown that the pepper sugar transporter genes had obvious spatiotemporal specific expression characteristics and exhibited variety-specific expression preferences. We focus on analyzing candidate genes that may be involved in fruit development and expansion. We further explore the response of pepper sugar transporters to adversity stress using a structural equation model. Finally, we found that the *MST*, *SUT*, and *SWEET* families are collectively involved in balancing pepper resistance to abiotic stress by coordinating the expression strengths of different family members. Our study may contribute to the functional study of pepper sugar transporter genes and create the prospect of utilizing sugar transporter gene resources to improve pepper variety.

## 1. Introduction

Sugar is not only a source of energy and a structural component but also a signaling factor involved in plant growth and development and stress response processes [1]. After the assimilation and fixation of CO_2_ in photosynthetic cells, different forms of sugars are transported and distributed as “carbon sources” within and among cells, from photosynthetic to non-photosynthetic tissues, and from aboveground to belowground. In photosynthetic cells, the sugars accumulated by plants during the day are temporarily stored in chloroplasts in the form of starch, which is hydrolyzed to glucose or maltose and transported out of the chloroplast at night, or synthesized into sucrose in the cytoplasm [2]. Sucrose is the major end product of photosynthesis and the main form of long-distance transport of plant sugars. Depending on the regulation of transport by sugar transporter proteins, plants can distribute energy from the green parts of the ground to the whole body to maintain their nutritional growth and reproductive development. Sugar transport and storage from source to sink organs are also closely related to crop yield, quality, and environmental adaptation [3]. Research on sugar transport will help us to better understand the underlying mechanisms of balanced growth and development in plants and their adaptation to adversity.

The exchange and conversion of monosaccharides between organelles such as chloroplasts and vesicles or between different cells is controlled by monosaccharide transport proteins (MSTs). Based on their substrate specificity and sequence characteristics, *MSTs* were further classified into seven subfamilies, including sugar transport proteins (*STP/HT*), early response to dehydration-6/sugar facilitator proteins (*ERD-6/SFP*), polyol monosaccharide/polysaccharide transport proteins (*PMT/PLT*), plastic glucose-like transport proteins (*pGlcT*), vesicular glucose transport proteins (*vGT*), glycosome monosaccharide transporter protein (*TMT*), and inositol transporter protein (*INT/ITR*) [4,5].

During long-distance transport, sucrose transporters load sucrose from mesophyll cells (source) to phloem and unload sucrose from the phloem of the “sink” tissue to assist sucrose entry into the sink cells [6]. The main plant sucrose transporters include *SUT* and *SWEET*, and amino acid sequence analysis reveals that plant *SUT* sequences are highly conserved, and all of them contain an MFS_2 (Pfam13347) structural domain with 12 conserved transmembrane structures. All plant *SWEET* proteins contain seven transmembrane structures with two MtN3_slv (Pfam03083) structural domains [7].

In recent years, the functions of plant sugar transporters in vegetative and reproductive growth, organ development, yield and quality formation, and response to adversity stress have been gradually revealed. In *Arabidopsis*, *AtSUC2* and *AtSUC4* were found to regulate plant abiotic stress tolerance through sucrose signaling, and *AtSUC3* strongly responded to tissue and organ mechanical damage [8,9]. *ZmSUT2* and *ZmSUT4* are closely related to grain yield traits in maize [10]. In cucumbers, *CsSUT1* controls flower development by regulating the sugar supply [11]. *SWEET* proteins function in sugar efflux. It was found that watermelon *ClVST1*, tomato *SlSWEET15*, and cucumber *CsSWEET7a* unload sucrose from the phloem to promote fruit growth and development [12,13,14]. In *Arabidopsis*, *SWEET11*, *SWEET12*, and *SWEET15* together regulate sucrose transport from the testa to the embryo via the endosperm [15]. In cotton [16], apple [17], banana [18], and tea [19], *SWEET*-mediated sugar accumulation in vacuole was found to be important for fruit ripening and plant resistance to cold, drought, and salt stress. *MSTs* that regulate the transport supply of monosaccharides can potentially contribute to crop yield and food quality. In rice, *OsMST4* [20] and *OsMST6* [21] are involved in seed development during grain filling (storage stage), and *OsGMST1* may play a direct or indirect role in salt stress tolerance in rice [22]. In *Arabidopsis*, *AtSTP13* enhances resistance to gray mold through sugar uptake [23], and *AtSTP8* is involved in host sugar transmembrane transport to the pathogen [24]. Overexpression of *AtTMT1* and *AtTMT2* genes can increase the seed biomass of *Arabidopsis thaliana* [25]. In grapes, monosaccharide transporters are the most important transporters. In grapevine (*Vitis vinifera*), the *VvHT3*, *VvHT4*, *VvHT5*, and *VvHT6* promoted sugar accumulation in fruit during the ripening stage [26]. The expression of *ClTST2* in watermelon increased the sweetness of watermelon pulp [27]. 

*Capsicum* is widely cultivated worldwide for the uniqueness and value of fruit. Explorations on sugar transport in pepper have been carried out one after another. It has been found that sugar transport is closely associated with the growth failure of pepper hybrid progeny [28]. In terms of quality improvement, *Actinomycetes* have been shown to enhance fruit flavor of pepper fruits by promoting sugar transport [29]. Genome-wide identification and characterization of *Capsicum* sugar transporters have not been carried out in depth. To further investigate the function and mechanism of *Capsicum* sugar transporters and to facilitate variety improvement, it is necessary to systematically identify and characterize pepper sugar transporter genes.

Through the 3D architecture of the pepper genome [30], this study characterized the compositional status of the pepper sugar transporter family in detail. *MST*, *SUT*, and *SWEET* were comprehensively analyzed in terms of physicochemical properties, chromosomal localization, gene structure, and phylogenetic relationships. The potential functions of pepper sugar transporters were analyzed by mining transcriptomic and proteomic data of different fruits and leaves. This research provides useful information for in-depth exploration of the physiological significance of sugar transporters in pepper breeding and the molecular mechanisms in fruit development.

## 2. Results

### 2.1. Identification of Gene Family Members

Overall, 3 *SUT*, 29 *SWEET*, and 50 *MST* members were identified in pepper (*Capsicum annuum* L.), which were renamed according to their location on the chromosomes (Supplementary Table S1). Sequence analysis revealed that each *SUT* sequence contained an MFS_2 (PF13347.6) structural domain. The amino acid sequences of the *SWEET* family member genes all contained two MtN3_slv (PF03083.16) structural domains. *MST* family members have MFS_1 (PF07690.16) and Sugar_tr (PF00083.24) domains, which have a large degree of sequence overlap.

In the *SUT* family, all the amino acid sequences are above 500 amino acids in length, the longest being *CaSUT03* with 611 amino acids. The molecular weight of SUT proteins ranges approximately from 54.27 KDa (Ca*SUT*1) to 65.55 KDa (Ca*SUT*3), with an average molecular weight of 58.20 KDa, and the isoelectric point values ranging from 6 to 9.32.

The shortest sequence of the SWEET family is CaSWEET02 with 186 amino acids, and the longest sequence is CaSWEET03 with 815 amino acids. The molecular weight of SWEET proteins approximately ranges from 21.00 KDa (Ca*SWEET*02) to 91.43 KDa (CaSWEET03), with an average molecular weight of 31.95 KDa, and an isoelectric point value ranging from 5.33 to 9.76. The shortest sequence of the MST family is CaMST35 with 117 amino acids, and the longest sequence is CaMST30 with 1262 amino acids. The molecular weight of MST protein ranges from 12.31.00 KDa (CaMST35) to 141.20 KDa (CaMST30), the average molecular weight is 54.55 KDa, and the isoelectric point value ranges from 4.89 to 9.63 (Supplementary Table S2).

**Figure 1:**
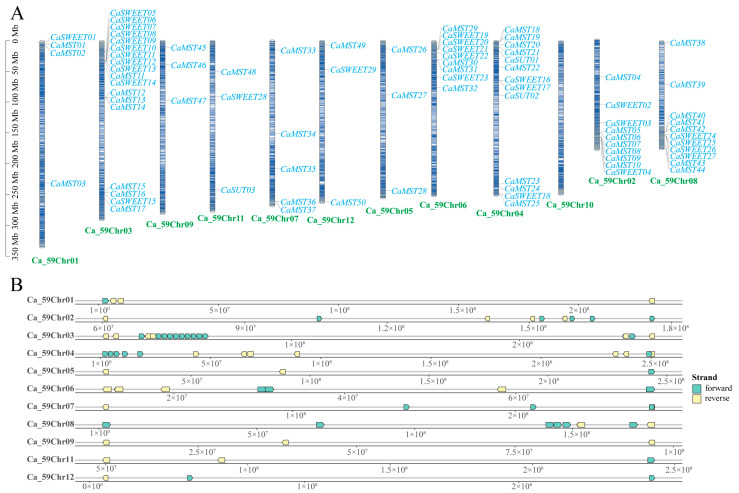
Distribution of the sugar transporter on the chromosome in pepper (*Capsicum annuum*). (**A**) All sugar transporter members were located on chromosomes. (**B**) Distribution of genes on the double strand. The chromosome numbers and sizes (Mb) are indicated at the bottom of each chromosome, respectively.

In *Capsicum*, the sugar transporter gene is distributed on 11 chromosomes (Figure 1A). *SUT* was distributed on chromosomes 4 and 11, and *SWEET* and *MST* were distributed on all 11 chromosomes, with a more concentrated distribution of the *SWEET* family gene. Chromosome 3 has the largest distribution of family genes, and most of the three family genes are located on the positive chain of chromosomes (Figure 1B).

### 2.2. Sequence Analysis and Gene Synteny

All identified family members were examined in terms of gene structures and amino acid sequence motifs. In the *MST* gene family (Supplementary Figure S1A), all members contain 4 or more motifs except *CaMST1*, *CaMST35*, and *CaMST39*. Among them, *CaMST1* contains only motif 5, and *CaMST35* contains only motif 7, which may be important for the functional formation of unique proteins. Most sugar transporter gene family members have more than three exons. Promoter sequence (2000 bp upstream of ATG) analysis revealed that the family member contains several cis elements related to environmental stress as well as development. Members of the same taxon on the phylogenetic tree have roughly similar motif composition and conserved structural domains, indicating that they are highly conserved. At the same time, members of different branches have independent motif composition and functional domains. As with *CaMST2/6/24/22/38/14/12/26/33/27/45*, they are divided within the same branch and have all ten motifs, whereas *CaMST1/35* has only one motif. And *CaSWEET6/10/9/8/11/12/20* show high similarity in motif composition. Meanwhile, the gene members of all three families contain more than one functional structural domain, suggesting that they may form a complex functional differentiation (Supplementary Figure S1A–C).

Different phylogenetic trees were constructed using protein sequences from *Capsicum*, *Arabidopsis*, and rice to study the evolution of sugar transporter protein homologs in different plant species. Phylogenetic analysis showed that the *Capsicum MST* gene family proteins can be divided into seven branches. *pGIcT* and *VGT* are the smallest subfamilies of the *Capsicum MST* gene family, both containing only two members (Supplementary Figure S2A). Based on the phylogenetic analysis of protein sequences from several species, the *SWEET* gene family was divided into four branches [31]. *Capsicum SWEET* gene family proteins are distributed in all four branches, with the largest number of branch III members, which are mainly responsible for sucrose transport (Supplementary Figure S2B). The *SUT* family genes in *Arabidopsis* were divided into three branches [32]. The corresponding *Capsicum SUT* gene family proteins are clustered in two branches, *CaSUT1* and *CaSUT2*, in the dicot-specific branch, which are type I transporter proteins, and *CaSUT3*, which is a type II transporter protein (Supplementary Figure S2C).

**Figure 2:**
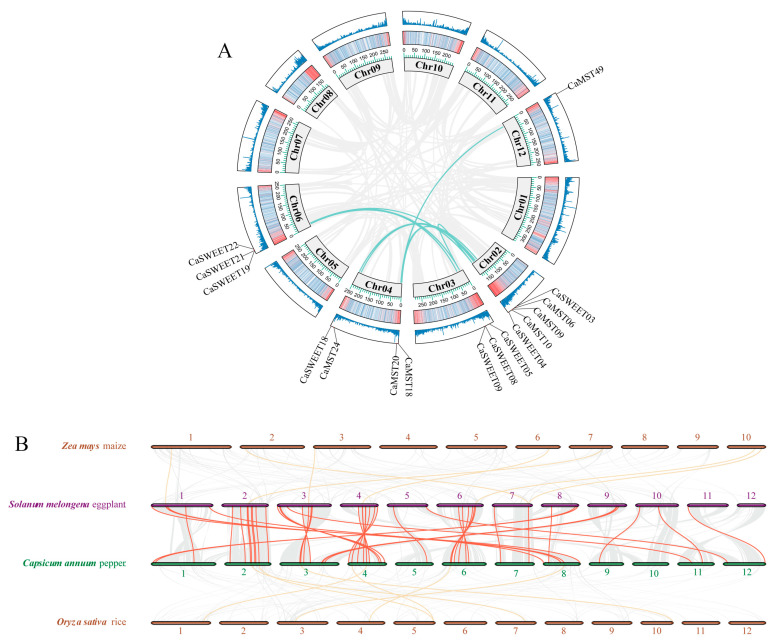
Duplication and synteny analysis of sugar transporter genes. (**A**) Schematic representations for the chromosomal distribution and inter-chromosomal relationships of pepper sugar transporter genes. (**B**) Synteny analysis of sugar transporter genes between pepper and eggplant, rice, and maize. The red lines highlight the synteny sugar transporter gene pairs.

The duplication event of three gene family members in pepper was analyzed. *Capsicum MST* gene family and *SWEET* family showed nine and seven gene doubling replications, respectively (Figure 2A). The synteny analysis among the four species, maize, rice, eggplant, and pepper, showed that the three sugar transporter genes showed high collinearity between eggplant and pepper and low between pepper and maize or rice (Figure 2B). It illustrated that the three gene family members were highly conserved among species of the same family. KA/KS calculations were also performed for tandemly duplicated genes. The results showed that all tandem duplicate genes within the *MST* gene family and the SWEET gene family were subjected to purifying selection (Supplementary Table S3).

### 2.3. Gene Expression Analysis among Different Varieties

Domestication and artificial selection may be a manifestation of environmental adaptation of species. To further explore the potential function of genes, we investigated the expression patterns of family gene members in the fruits of four wild and six domesticated pepper varieties, and for each variety, samples of seven stages were collected.

**Figure 3:**
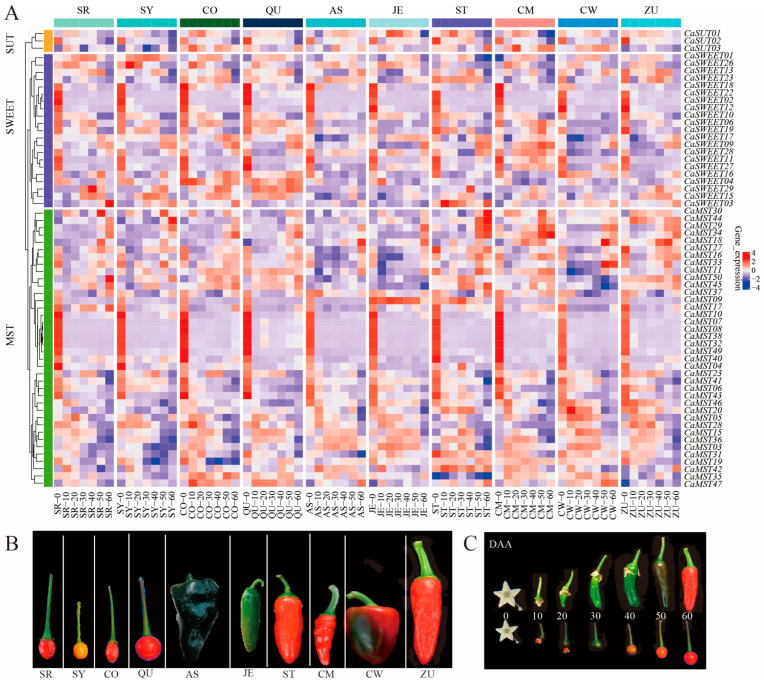
Heatmap of sugar transporter genes during different pepper fruits. (**A**) Expression profile of sugar transporter genes in six domesticated (AS, JE, ST, CM, CW, ZU) and four wild (SR, SY, CO, QU) varieties, (**B**) ten pepper varieties, (**C**) and pepper fruit developmental stages (0DAA, 10DAA, 20DAA, 30DAA, 40DAA, 50DAA, 60DAA; DAA: days after anthesis) [33]. The values in the image of heatmap are the averages of three biological replicates (log2(TMM + 1)). The images depicted in the manuscript are only representative of the sample’s appearance, and they are not to scale.

To understand the roles of *MST*, *SWEET*, and *SUT* genes in the fruit of pepper, we analyzed transcriptome sequencing data from ten pepper varieties at seven fruit developmental stages (0DAA, 10DAA, 20DAA, 30DAA, 40DAA, 50DAA, 60DAA; DAA: days after anthesis). The results showed that members of *MST*, *SWEET*, and *SUT* genes were differentially expressed at different developmental stages of pepper fruits. The expression level of *CaSUT1/3* was relatively stable at most of the fruit stages, and *CaSUT2* was highly expressed at the first stage of fruit and was least expressed at the seventh stage of fruit, and this expression pattern was consistent within the ten varieties (Figure 3A). *CaSWEET02/05/11/12/18/22* had the highest expression in the first stage of fruit development and maintained very low expression levels in the other six stages. *CaSWEET07* was not expressed in the seven developmental stages of most varieties and was only highly expressed in the seventh stage of a few varieties. *CaMST02/07/08/10/32/38/40* had the highest expression in the first stage of fruit development and maintained very low expression levels at the other six stages. *CaSWEET04/29* maintained high expression throughout the fruit developmental stages of the wild variety (CO and QU) relative to the domesticated variety. In contrast, *CaMST03/31/36* maintained higher expression throughout the fruit developmental stages of the domesticated variety relative to the wild variety (Figure 3B).

**Figure 4:**
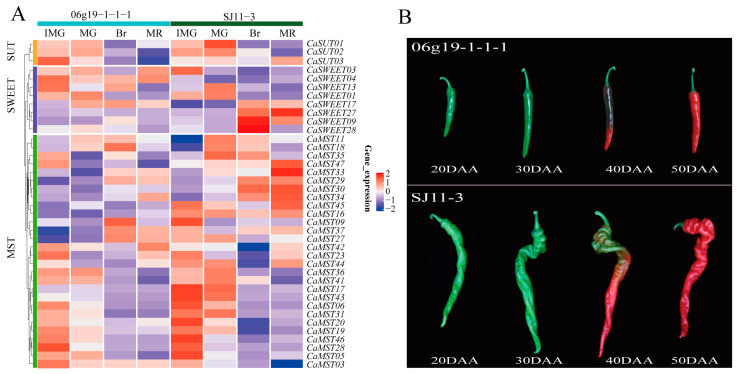
Expression profile of sugar transporter genes in SJ11-3 and 06g19-1-1-1. (**A**) Heatmap of sugar transporter genes and (**B**) pepper fruit developmental stages (20DAA, 30DAA, 40DAA, 50DAA; DAA: the day after anthesis) [34]. The values in the image of heatmap are the averages of three biological replicates. The images depicted in the manuscript are only representative of the sample’s appearance, and they are not to scale.

Fruit size and shape are important commodity traits of peppers. We investigated the expression characteristics of sugar transporter genes in pepper varieties (SJ11-3 and 06g19-1-1-1) with significantly different fruit weights, including four developmental stages (20DAA, 30DAA, 40DAA, 50DAA). The fruit of “SJ11-3” is long and screw-shaped, while the fruit of “06g19-1-1-1” is short (Figure 4). *CaSWEET09/28* was only highly expressed in the 40DAA phase of SJ11-3 and maintained a very low expression level in other stages. *CaSWEET27* was only highly expressed in the 50DAA phase of SJ11-3 and maintained a very low expression level in other stages. Most of the MST members maintained high expression levels in the 20DAA stage of fruits, and SJ11-3 was higher than 06g19-1-1-1. The expression characteristics of *SUT* members were similar in the two cultivars (Figure 4A,B).

**Figure 5:**
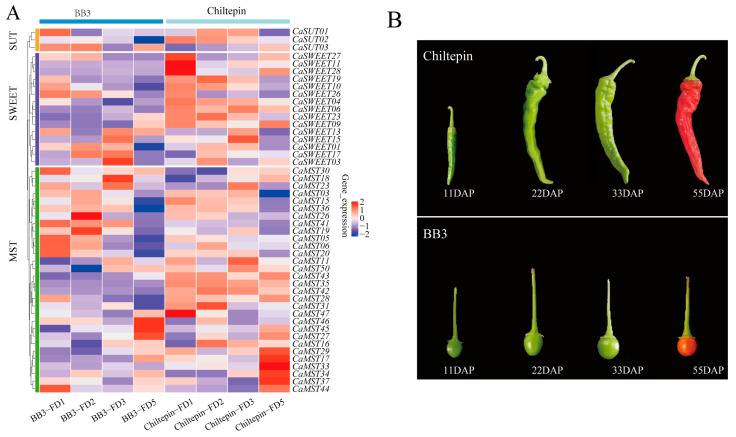
Expression profile of sugar transporter genes in BB3 and Chiltepin. (**A**) Heatmap of sugar transporter genes and (**B**) pepper fruit developmental stages (11DAP; 22DAP; 33DAP; 55DAP; DAP: the day after pollination) [35]. The values in the image of heatmap are the averages of three biological replicates. The images depicted in the manuscript are only representative of the sample’s appearance, and they are not to scale.

Spiciness is an important flavor characteristic of pepper. We observed the expression of family genes in pepper fruits with different pungency, including the four developmental stages (11DAP; 22DAP; 33DAP; 55DAP; DAP: the day after pollination). BB3 has a slight spiciness, whereas Chiltepin is much hotter than BB3 [35]. Overall, sugar transporter genes tend to occur in the first three stages of fruit development. *CaMST26* was expressed only in the 22DAP phase of BB3, and *CaMST27/45/46* was the highest in the 55DAP phase of BB3. *CaSWEET11/27/28* and *CaMST47* were the highest in the 11DAP phase of Chiltepin and maintained a very low expression level in other stages. *CaMST29/17/33/34/37* was the highest expression in the 55DAP phase of Chiltepin and maintained a very low expression level in other stages. This expression characteristic may be related to the accumulation of spiciness in peppers (Figure 5A,B).

**Figure 6:**
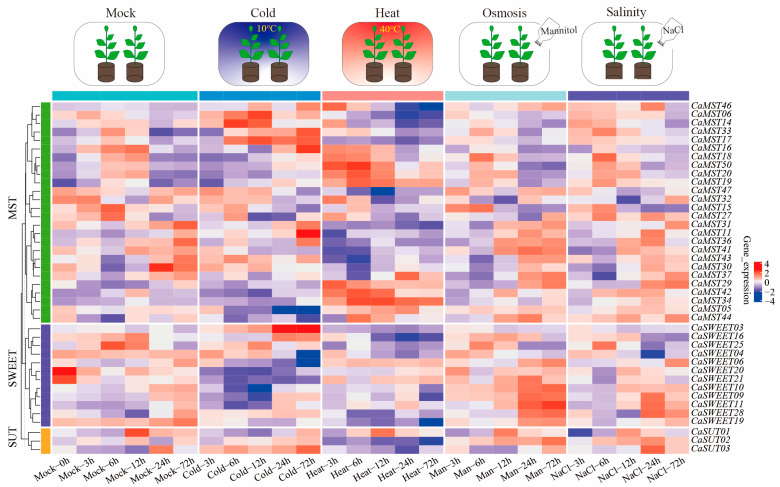
Heatmap of transcriptome expression of sugar transporter genes of pepper (CM334) leaves under abiotic stress (heat, cold, salinity, and osmotic) [36]. Each differently colored rectangle of the heatmap represents one gene. The values in the image are the averages of three biological replicates. Leaves were harvested at 3, 6, 12, 24, and 72 h after treatment. Plants (at the six-true-leaf stage) were treated with 50 mL of a 400 mM solution.

Sugar transport is a strategy for plants to adapt to their environment. We further observed the expression patterns of family member genes in pepper leaves at the six-true-leaf stage of cultivar CM334, under different stress treatments (heat, cold, salinity, osmosis; 3, 6, 12, 24, and 72 h). The results showed that *CaSWEET20/21* and *CaMST30* had the highest expression levels under a normal growth environment, and the expression levels decreased rapidly after stress treatment. *CaSWEET03* and *CaMST11/16/17/18* had the highest expression levels only after 24 h of low-temperature stress. The highest expression level of *CaMST50* was maintained after 6 h of high-temperature stress, and the expression level was very low under treatment. *CaSUT02* and *CaSWEET11* were highly expressed under salt stress and osmotic stress but did not respond significantly to other stress treatments. By comparing the differential expression with the control, we explored the overall expression trend of pepper sugar transporter genes under abiotic stress conditions (heat, cold, salinity, osmosis) (Supplementary Figure S3). Overall, the sugar cotransport genes showed a positive response pattern under stress conditions (Figure 6).

### 2.4. Differential Expression Analysis

To further understand the expression characteristics and possible mechanisms of action of MST, SWEET, and SUT genes in pepper, we analyzed transcriptomic and proteomic data of fruits of two pepper varieties with significant differences in size. In the experiment, pepper fruits were classified into four developmental stages (20DAA, 30DAA, 40DAA, 50DAA) based on fruit color and developmental degree. Different from the conventional process of transcriptome data analysis, PCA analysis was performed here with the transcriptome data of three gene family members as a way to detect the degree of inter- and intra-group variability. As shown in Supplementary Figure S4A, 24 samples could be separated into the first two PCs, accounting for 83.48% of the total variability. PC1 accounted for 65.47% of the variability, and PC2 accounted for 18.01% of the variability. In pepper varieties SJ11-3 and 06g19-1-1-1, fruit 40DAA stage and 30DAA stage had the maximum distance in the PC1 dimension, and the most important gene in this dimension was CaMST36. The fruit 40DAA stage and 20DAA stage of pepper variety SJ11-3 had the maximum distance in the PC2 dimension, and the fruit 30DAA stage and 50DAA stage of pepper variety 06g19-1-1-1 had the maximum distance in the PC2 dimension, and the most important gene in this dimension was CaSWEET09 (Supplementary Figure S4B).

The results of the Wayne diagram analysis showed that 38 of the three families of genes (SUT:3, SWEET:29, MST:50) were expressed in all the stages, and six genes, CaSWEET02/05/07 and CaMST01/07/21, were not expressed in all the stages (Supplementary Figure S4C,D).

Based on the results of sample hierarchical clustering and PCA analysis, we performed differential expression analysis of the family genes in different fruit developmental stages of the two varieties. *CaSWEET09/17* and *CaMST29* were significantly down-regulated, and *CaMST06/28* was significantly up-regulated in SJ11-3 pepper during fruit development from 20DAA to 40DAA stage, with more than five-fold changes in gene expression levels (Supplementary Figure S5A). *CaSWEET09* was down-regulated with the greatest fluctuation, and *CaSWEET01* was significantly up-regulated during the process from 30DAA to 40DAA stage of the fruit (Supplementary Figure S5B). Differential expression analysis of 06g19-1-1-1 pepper fruits at 50DAA and 30DAA stages showed that *CaMST36* fluctuated with the greatest change (Supplementary Figure S5C). Differential expression analysis at 40DAA and 30DAA stages showed that *CaMST15/36* and *CaSWEET01* were significantly down-regulated at 40DAA stage, and *CaMST09/29* and *CaSWEET09* were significantly up-regulated at 40DAA stage (Supplementary Figure S5D). Overall, during the fruit development of the two pepper varieties, members of the three gene families experienced the largest expression fluctuation from 30DAA stage to 40DAA stage.

Gene set enrichment analysis (GSEA) was used to further explore the overall expression of the members of the three gene families during fruit development. When analyzing the expression of genes in samples differing in traits, traditional enrichment analysis methods yield a batch of differentially expressed candidate genes. These genes may be closely related to the formation of traits in the samples. They are often categorized into a particular metabolic pathway. This approach ignores the overall expression trends of genes and incorporates subjective screening criteria for differential genes. GSEA analysis sorts genes from largest to smallest according to the ploidy of differential expression of the genes in the two sets of samples. After sorting the list of genes, its top can be seen as up-regulated differential genes and its bottom as down-regulated differential genes. Considering a gene family as a set of genes, it is analyzed whether the family member genes are enriched at the top or the bottom of this sorted list. If they are enriched at the top, in general, the gene set is up-regulated and, conversely, if they are enriched at the bottom, they are down-regulated. The results showed that, with the expansion of fruit development, the members of the three gene families as a whole showed a down-regulation trend with fruit development and expansion (Supplementary Figure S6A–D).

### 2.5. Weighted Gene Co-Expression Network Analysis 

**Figure 7:**
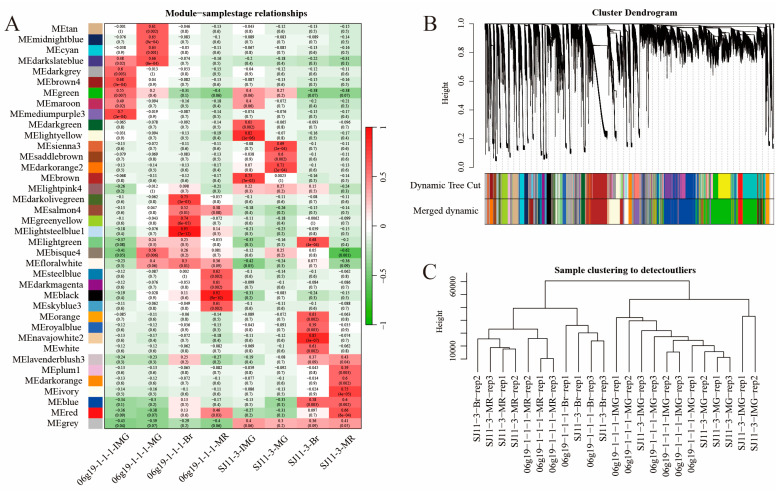
Identification of modules associated with the development periods. (**A**) Heatmap of the correlation between the module eigengenes and development periods of pepper fruit. (**B**) Dendrogram of all expressed genes clustered based on the measurement of dissimilarity (1-TOM). The color band shows the results obtained from the automatic single-block analysis. (**C**) Hierarchical clustering among all repeat samples based on all expressed genes.

Weighted gene co-expression network analysis was performed using transcriptional data for all genes, which were divided into 38 modules (Figure 7A). The modules with the highest correlation with the four developmental periods of pepper fruits were screened, and the distribution of gene family members was viewed in the modules. For the four periods of fruit development (20DAA; 30DAA; 40DAA; 50DAA) of the SJ11-3 pepper variety, we selected “brown”, “darkorange2”, “blue”, and “ivory”, and for the four periods of fruit development of the 06g19-1-1-1 pepper variety, the modules “mediumpurple3”, “green”, “lightsteelblue1”, “steelblue” were selected, respectively (Figure 7B,C).

Focus was placed on genes with an absolute value of correlation with the module greater than 0.8 and with the trait (fruit developmental stage) greater than 0.2. *CaMST04/06/09/17/22/43* were located in the highlighted regions during the 20DAA period of SJ11-3 pepper fruits. No family genes were labeled at the 30DAA period. At the 40DAA period, *CaMST29/30* were labeled, and at the 50DAA period, *CaMST27* was labeled (Supplementary Figure S7A–D). In the 06g19-1-1-1 variety, *CaSWEET 24/25* and *CaMST38* were highlighted at the fruit 20DAA stage. At the 30DAA period, *CaMST41* is marked. At the 40DAA period, *CaSWEET15* was labeled, and *CaMST12* was marked in the 50DAA period (Supplementary Figure S7E–H).

### 2.6. PPI

Protein–Protein Interaction Networks (PPIs) are composed of proteins that interact with each other to participate in various aspects of life processes such as biological signaling, regulation of gene expression, energy and material metabolism, and cell cycle regulation. Protein network interactions were analyzed for three sugar transporter gene family members. Eighty-two protein sequences were inputted, and the interaction network information of 12 protein sequences was obtained. The results showed that CaSUT01/02 and CaSWEET18 possessed the highest betweenness centrality. The SWEET family members in the network were CaSWEET01/02/15/24/26/289, and the MST family members were CaMST27 and CaMST45.All the SUT family members were in the protein interactions network (Supplementary Figure S8).

### 2.7. Mantel Test Analysis

Currently, there is a lack of reports on the interaction between sugar transport genes at the family level. Most research models focus on identifying correlations between pairs of genes. In terms of data structure, the expression data of two genes in different samples can be viewed as “columnar” data. However, our aim is to investigate the interactions between gene families across different samples, which represents a type of “matrix” data. Traditional correlation analysis methods (Pearson, Spearman, and Kendall) are only capable of handling the correlation between two columns of data and are inadequate when confronted with the correlation between two matrices.

**Figure 8:**
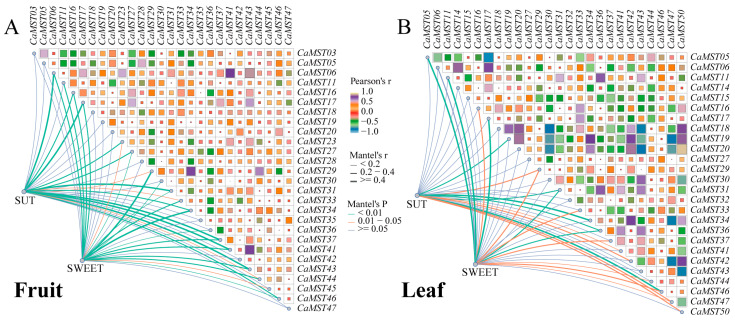
Mantel test analysis of the three sugar transporter families. (**A**) Correlation between *SUT*, *SWEET*, and *MST* gene families based on expression data of pepper fruit in different varieties. (**B**) Correlation between *SUT*, *SWEET*, and *MST* gene families based on expression data of pepper leaf in CM334 under stresses. Edge width corresponds to Mantel’s r statistic for the corresponding distance correlations, and edge color denotes the statistical significance. Pairwise comparisons of intestinal *MST* family members are shown, with a color gradient denoting Pearson’s correlation coefficient.

Mantel test analysis was used to further explore the interaction between the three gene family members in pepper fruit development. Genes expressed in all samples were selected for correlation analysis. The results showed that *CaMST06/41/43* was positively correlated with *SUT* family members (r > 0.5, *p* < 0.01), and *CaMST16/29/34* was significantly positively correlated with *SWEET* family members (r > 0.3, *p* < 0.01) (Figure 8A). In pepper leaves under different treatments, correlation analysis showed that *CaMST31/34/46* was significantly positively correlated with *SUT* family members (r > 0.3, *p* < 0.01), and *CaMST05/11/17* was significantly positively correlated with *SWEET* family members (r > 0.4, *p* <0.01) (Supplementary Table S4). Generally, the *SWEET* family had a greater effect on *MST* family members (Figure 8B). 

### 2.8. PLS-SEM Analysis 

**Figure 9:**
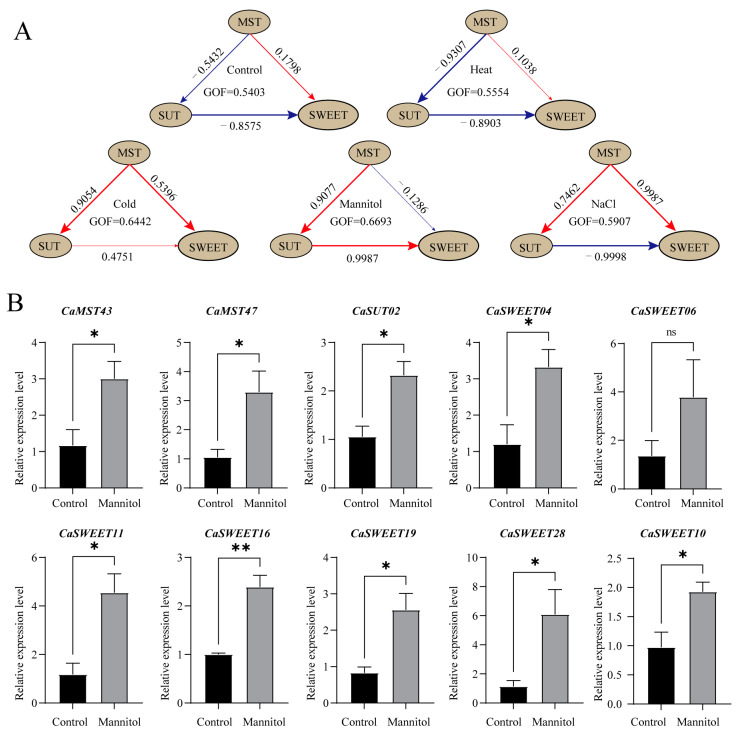
PLS-SEM analysis of pepper sugar transporters and the expression of manifest variables. (**A**) PLS-SEM analysis of pepper sugar transporters. The brown ovals represent latent variables, and the number on the arrows indicates the path coefficient. (**B**) The expression of manifest variables. Genes expressed in all samples (leaf) under treatments were selected for PLS-SEM analysis (Partial Least Squares Structural Equation Modeling). Each gene is considered as a manifest variable. The 10 genes with the highest expression levels under mannitol treatment were selected for qPCR analysis (*: *p* ≤ 0.05; **: *p* ≤ 0.01; ns: *p* > 0.05). The values in the image are the averages of three biological replicates. Plants (at the six-true-leaf stage) were treated with 400 mM mannitol solution, and water was applied to the corresponding control group. Leaves were harvested at 24 h after treatment.

Genes expressed in all samples (leaf) under treatments were selected for PLS-SEM (Partial Least Squares Structural Equation Modeling) analysis to further explore the interaction between sugar transport families. The results showed that, under the normal growth environment simulated in the control group, the *MST* family showed a negative effect relationship for the *SUT* family, a positive effect relationship for the *SWEET* family, and *SUT* family had a negative effect relationship for the *SWEET* family (Figure 9A). Under salt stress and low temperature, the *MST* family showed a positive effect relationship with the *SUT* family. However, the results of the high-temperature treatment showed little difference from the normal conditions. At the same time, positive effects were shown among the three families under low-temperature stress, indicating that the sugar transport genes had stronger responses to low-temperature stress. Interestingly, under the conditions of osmotic stress simulated by mannitol, the relationship between the three families was exactly opposite to that under normal conditions. To further verify the expression characteristics of sugar transporter genes under a stressful environment, we selected 10 genes with the highest expression levels under mannitol treatment for qPCR analysis. The results showed that, except for *CaSWEET06*, the expression levels of the other nine genes were significantly higher than those of the control group after stress treatment. These results are generally consistent with transcriptome data, suggesting that there may be a complex synergistic relationship between sugar transporter genes in response to different stress environments (Figure 9B).

## 3. Discussion

The important role of sugar in plant growth and development, signaling, and resistance to adversity stress depends on the distribution, uptake, storage, and transport by sugar transporters [37]. Due to their importance, genome-wide analyses of sugar transporters have been carried out extensively in many sequenced species [38]. In pepper, relevant studies have not been reported. In this study, 54 *MST*, 29 *SWEET*, and 3 *SUT* were identified in the pepper genome, and the expression properties and structures of the gene family members were predicted.

We have initially investigated the promoter structures, protein physicochemical properties, and phylogeny of the sugar transporters. The isoelectric points of proteins are related to their stability and aggregation tendency and can provide some information about their structure and function. In pepper, the length range of sugar transporter proteins was 117-1262 AA, isoelectric point range was 4.89–9.76, and protein molecular weights were 12.31–141.22 kDA. Sugar transporters are mostly transmembrane proteins [39]. The pH value of the two sides of the lipid membrane may differ greatly. *Capsicum* sugar transporters span a wide range of isoelectric points, both acidic and basic. This may partially mirror the complex working environment that sugar transporters cope with. A total of 64% of the pepper sugar transporter proteins contained five or more exons. In some studies, genes with fewer or no introns are thought to be expressed at higher levels in plants [40]. The presence of introns makes it necessary for eukaryotic cells to expend a large amount of energy and nucleotides to synthesize, shear, and degrade genes during gene expression, which undoubtedly increases the burden on the survival of eukaryotic cells [41,42]. The fact that the *Capsicum* sugar transporter contains fewer introns may make the gene structure more compact and therefore increase the efficiency of transcription. To respond to various stresses promptly, genes have to be activated quickly, and a compact gene structure with fewer introns would facilitate this [43,44]. Compact gene structure of the pepper sugar transporters may be consistent with the properties of structural genes that distinguish them from regulatory genes such as transcription factors and signal transduction proteins. Some studies have indicated that sugar transport genes play a role in plant response to abiotic stresses such as drought and cold injury. The overexpression of sugar transport genes often leads to an increase in the content of soluble sugars in leaves, which enhances leaf resistance to osmotic stress. Based on these findings, it is hypothesized that the *Capsicum* sugar transporter gene may also possess similar stress-resistant functions [45,46].

A motif refers to a basic structure that constitutes a characteristic sequence. It is generally considered to be a conserved sequence that possesses a biological function, and may contain specific binding sites, or a sequence segment with commonality involving a particular biological process. Motif often implies a certain protein structural domain, which is understood as a sequence commonality of a broad class, and is usually also associated with a specific function [47]. The results of motif and domain analyses of the *Capsicum* sugar transporter family reflect a high degree of consistency, with the motifs and domains of the different subfamily proteins clearly distinguishing them from the other subfamilies, suggesting that they may have their specific functions. Plant sugar transport proteins are primarily membrane proteins. They can cross cellular, vesicular, mitochondrial, and other plasma membranes. Previous studies have reported that sugar transporter proteins of the same family are usually divided into several isoforms. They have distinct structural differences, which may be related to their substrate specificity and affinity. Meanwhile, promoter analysis revealed that members of the *Capsicum* sugar transporter gene family contain a large number of cis elements related to plant growth and development, stress response, and signal transduction. Phylogenetic analyses also showed that most of the *Capsicum* sugar transporter protein subfamilies share close homology with their corresponding members in rice and *Arabidopsis thaliana*, and thus, it was hypothesized that the roles of the *Capsicum* sugar transporters may be highly coordinated and widespread.

The formation and expansion of a gene family depends on the duplication of genes, and these duplicated genes contribute to the evolution of genes with new functions, as well as to the different distribution patterns of genes on chromosomes [48,49]. Members of the *Capsicum* sugar transporter gene family are widely distributed on all chromosomes except chromosome 10, with most of the *MST* and *SWEET* member genes arranged in clusters at both ends of the chromosome. A high degree of tandem duplication was found among these genes in the *PLT/STP/ERD* subfamily of the *MST* family and TypeBI/TypeAII of the *SWEET* family. It can be inferred that the pepper transporter gene has undergone segmental duplication and tandem duplication and has formed multiple subfamilies and members along with phylogenetic evolution. The Ka/Ks analysis results of these tandem duplicated genes were all less than 1, which may indicate that the *Capsicum* transporter genes were mainly subjected to purification selection during evolution.

Similar selection events may have influenced the presence, functional divergence, and convergence of sugar transporter proteins in monocotyledonous and dicotyledonous plants [50]. The analysis of collinearity between species can reveal the distribution or arrangement of homologous genes within different species. This may help us to understand the origin and evolutionary details of genes [51]. In a collinearity analysis conducted between the four species, it can be seen that sugar transporters differ significantly between two monocotyledonous plants (rice and maize) and two dicotyledonous plants (pepper and eggplant) (Figure 2B). The collinearity of sugar transporter genes between *Solanaceae* species was much higher than that between *Poaceae* species. Moreover, the pepper sugar transporter proteins were more often in the same clustering branch as the *Arabidopsis* sugar transporter proteins, and it suggested that the pepper sugar transporter possesses higher sequence similarity to the *Arabidopsis* sugar transporter relative to rice.

The evolution of gene families occurs as family members respond to life activities. In a complex series of expression-regulated processes, sugar transporter proteins control the distribution of sugar transport in “sink” (roots, seeds, and fruits) and “source” (green tissues) [52]. This study focuses on changes in the expression of sugar transporters in the fruits of *Capsicum*. We first analyzed transcriptomic data from 10 varieties of *Capsicum* during seven developmental stages to explore the potential function of sugar transporter genes and further verify the robustness of identification of gene family members.

Expression of sugar transporter genes in fruit regulates the developmental process and commercial quality of fruits. The *SWEET* gene in tomatoes regulates the ratio of glucose to fructose in fruit [53]. *PbTMT4* in pear mediates vesicular sugar transport and strongly affects sugar accumulation in fruit [54]. *MdSUT4* negatively regulates sugar accumulation in apple fruit [55]. Pepper is a typical non-respiratory jumping fruit, and sugar transporter genes may also be involved in the regulation of pepper fruit ripening [56]. RNA-seq data showed that most of the sugar transport members of *Capsicum* were significantly expressed during early fruit development. The expression patterns of the *SUT* family members varied but remained largely consistent among varieties. *CaSUT1* maintained low expression during early and late fruit development and high expression during the intermediate period. In contrast, *CaSUT3* maintained high expression in early and late fruit development and low expression in the intermediate period, while *CaSUT2* transcript expression levels gradually became lower with fruit development. *CaSWEET02/05/11/12/18/22* and *CaMST02/07/08/10/32/38/40* showed similar patterns in fruit development, with high expression at the first stage, while maintaining very low expression levels at the other six stages. *CaSWEET07* was highly expressed in the seventh stage of fruit development of a few varieties, and *CaSWEET21* was highly expressed in the first stage of fruit development of a few varieties. This may be a reflection of the spatiotemporal specific expression characteristics of sugar transporter genes.

Fruit ripening and sugar accumulation is a complex regulatory process that requires the participation of many sugar transporters and sugar metabolism genes. To further explore the relationship between pepper sugar transporter genes and pepper fruit development, two pepper varieties with significant differences in fruit size and weight were selected, and transcriptomic and proteomic data from four developmental stages of fruit (20DAA, 30DAA, 40DAA, 50DAA) were jointly analyzed [34]. The results of PCA analysis using only transcriptome expression data of gene family members showed that samples from different varieties with different fruit developmental stages had clear separation and distance in PC1 and PC2 dimensions. This suggests that the pepper sugar transporter genes function stage-wise and specifically during fruit development and that this specificity is reflected among varieties. This may provide certain messages for variety selection and germplasm resource utilization. *CaMST36* and *CaSWEET09* were the two genes with the highest contribution in the PC1 and PC2 dimensions. They might play important roles in different developmental stages of pepper fruits. It was also found that six genes, *CaSWEET02/05/07* and *CaMST01/07/21*, were not expressed in all stages of the two pepper varieties, SJ11-3 and 06g19-1-1-1. This may be related to the tissue-specific expression of the genes. Differential expression analysis of the most divergent sample clusters revealed that *CaSWEET09/17* and *CaMST29* were significantly down-regulated and *CaMST06/28* were significantly up-regulated in SJ11-3 pepper during the progression of the fruit from the 20DAA stage to the 40DAA stage, a process that may have facilitated the rapid accumulation of sugar in pepper and the expansion of the fruit shape. The greatest change in *CaMST36* was observed from the 30DAA stage to the 50DAA stage of 06g19-1-1-1 pepper fruits. At this stage, the changes in size and shape of pepper fruits were over, and there was a shift from massive storage of primary metabolites to secondary metabolism accumulation, and *CaMST36* may have contributed to the generation and persistence of this change.

The functions and expression patterns of members in the same family are often similar, but there are special members of functional differentiation at the same time. Therefore, it is difficult to explain the role of the whole family in plant growth and development from the perspective of individual genes [57]. Here, GSEA and WGCNA analyses were used to summarize the performance of the entire family of genes during pepper fruit development. Genes were ranked in terms of their degree of differential expression (logFC) in two samples of pepper fruit developmental stages (not counting differential genes), and members of the pepper sugar transporter gene family were used as a gene set to determine whether the gene set tended to fall at the top or bottom of the ordered list [58]. In the four groups of differential analyses, most of the gene family members were below “0”. It is therefore hypothesized that the pepper sugar transporter gene family as a whole is progressively down-regulated as the fruit develops and expands, which may predict a gradual decrease in the intensity of the corresponding sugar transport.

The analysis method of artificially setting thresholds to screen differentially expressed genes may ignore those genes whose transcriptional expression levels do not fluctuate much but play an important role. WGCNA incorporates the expression levels of all genes, and the construction of a scale-free network through the TOM matrix allows for the screening of genes tightly associated with a sample or trait [59]. Based on the expression characteristics of the genes, the transcribed genes detected at all fruit developmental stages of two pepper varieties, SJ11-3 and 06g19-1-1-1, were classified into 38 modules, and members of the sugar transporter gene family were distributed in 25 of these modules. Eight modules with the highest correlation with the fruit development stage were selected as the screening objects for candidate “hub” genes, focusing on MM > 0.8 and GS > 0.2 genes. At the first stage of fruit development (20DAA), the candidate genes for 06g19-1-1-1 and SJ11-3 were *CaMST04/06/09/17/22/43* and *CaSWEET24/25*, *CaMST38*, respectively, which were higher than those at the other three stages of fruit development and differed between the two varieties. This may indicate that pepper fruit has a higher sugar transport intensity during the pre-developmental period and that the intensity of sugar transport varies between varieties for the same fruit developmental period.

The pervasive and complex linkage of different genes constitutes a vast network of interactions. Protein interaction network analysis of pepper sugar transporter family members revealed that *CaSUT01/02* and *CaSWEET18* possessed the largest betweenness centrality in the family, suggesting that they have a great influence on the pepper sugar transporter family and may be one of the central nodes of the complex regulatory network of sugar transporter. The results of correlation analysis showed that the degree of association among *MST*, *SWEET*, and *SUT* was inconsistent among the different varieties, and the correlations between different members of the same family and other families had obvious strong and weak variations, and the performance among genes was not homogeneous. This may be related to the complexity and breadth of the function of the pepper sugar transporter family. The varying yet uniform performance of members of the same family in different stages of fruit development may underlie the precise regulation of pepper sugar transport. 

SEM (Structural Equation Modeling) is a statistical analysis method used to study complex relationships among multiple variables [60]. The involvement of sugar transporter genes in plant response to adversity stress has been widely reported. Structural equation modeling analysis can reveal the basic situation of sugar transporter genes involved in the stress response of pepper. The results showed that different family members of pepper sugar transporter genes could coordinate their respective expressions to cope with different unfavorable environments. 

Plants can modulate sugar transport to increase tolerance to abiotic stresses. Sugar transporters play an important role in this. Sugar transporter genes are induced under drought, cold, or high salt stress, which improves the redistribution of sugars in the plant, which in turn leads to plants that exhibit higher levels of resistance [61]. However, the mode of action among plant sugar transporter families has been reported in few studies. Under stress conditions (heat, cold, salinity, and osmotic), *Capsicum* sugar transporter families showed rich interaction details. The results of structural equation modeling (PLS-SEM) showed that, under high temperature stress, the *MST* family of *Capsicum* showed a strong positive interaction with the *SUT* family. Under low-temperature stress, the *MST* family of *Capsicum* exhibited a strong negative interaction relationship with the *SUT* family. Under osmotic stress, the *Capsicum SUT* family exhibited a strong positive interaction relationship to the *SWEET* family, whereas under salt stress conditions, the pepper *SUT* family exhibited a strong negative interaction relationship to the *SWEET* family. This mode of action between sugar transporters that changes with the stress environment may suggest a sophisticated regulatory mechanism. Meanwhile, we found that, under high temperature stress, the mode of action among the *Capsicum* sugar transporters remained essentially unchanged relative to the control. However, under osmotic stress, a diametrically opposite mode of action was observed among the *Capsicum* sugar transporters. This suggests that the interactions among pepper sugar transporters may be closely related to the osmotic environment within the cell. This is consistent with the conclusion that sugar transporter genes can promote the accumulation of cellular soluble sugars to maintain osmotic homeostasis [62]. This phenomenon may be related to the regulation of osmotic pressure in plant cells by sugar transporter genes [7]. However, more about the regulation of interactions between pepper sugar transporters still needs to be explored further.

## 4. Materials and Method

### 4.1. Sequence Download

Pepper (*Capsicum annuum* L.) genome sequence information was obtained from NCBI (https://www.ncbi.nlm.nih.gov/bioproject/PRJNA788020, accessed on 11 September 2023). The *Arabidopsis* thaliana protein sequences were downloaded from TAIR (https://www.arabidopsis.org/, accessed on 13 September 2023), and the rice (*Oryza sativa*) protein sequences were downloaded from the China Rice Data Center (http://www.ricedata.cn/gene/index.htm, accessed on 15 September 2023). The Hidden Markov Model (HMM) file of the conserved structural domains of sugar transporter proteins was downloaded from Pfam (http://pfam.sanger.ac.uk/) [63]. Raw data for transcriptome sequencing of twelve pepper varieties for seven fruit developmental periods were obtained from NCBI (https://www.ncbi.nlm.nih.gov/Traces/study/?acc=PRJNA694629&o=acc_s%3Aa, accessed on 1 October 2023). Transcriptome data for four developmental periods of two pepper varieties with differing fruit types were obtained from NCBI (https://www.ncbi.nlm.nih.gov/Traces/study/?acc=PRJNA485468&o=acc_s%3Aa, accessed on 5 October 2023).

### 4.2. Identification of Gene Family Members

The whole genome protein sequences of pepper were scanned by hmm-search, E-values of <1 × 10^−5^ were set for filtering, and the filtered pepper protein sequences were used to construct a new species-specific HMM and searched again. The published proteins of rice and *Arabidopsis* family members in NCBI were used as query sequences, and E-values of <1 × 10^−10^ were set to blast the pepper genome protein database. The ensemble of the Pfam search and blast results was taken, and finally, NCBI-CDD was used (http://www.ncbi.nlm.nih.gov/Structure/cdd/wrpsb.cgi, accessed on 5 October 2023) to identify proteins containing the target structural domains as members of gene families. Protein physicochemical properties of gene family members were analyzed through the ExPASy proteomics server (http://ca.expasy.org/prosite/, accessed on 5 October 2023). 

### 4.3. Sequence Analysis

The upstream (2000 bp) of the initiation codon (ATG) was used as a promoter, which was analyzed via the online website PlantCARE website (http://bioinformatics.psb.ugent.be/webtools/plantcare/html/, accessed on 7 October 2023) for promoter cis element analysis. Conserved motifs in the protein sequences were identified through the online program MEME (http://meme.nbcr.net/meme/intro.html, accessed on 19 October 2023), with the optimal width of each motif ranging from 8 to 250 residues and the maximum number of motifs set to 10.

### 4.4. Phylogenetic Analysis

Protein sequences were compared by MEGA (version 11.0), and phylogenetic trees were constructed using the maximum likelihood method [64]. The conformational tree model was adopted with “Poisson correction” and “pairwise deletion” options, and branch reliability was evaluated through 1000 iterations of the bootstrap test. Images were drawn via the ITOL website [65].

### 4.5. Gene Chromosome Distribution and Duplicating Analysis

Gene chromosome distribution information was displayed by TBtools v2.026 software [66]. Gene family duplication event analysis by Multiple Collinearity Scan toolkit (MCScanX) [67]. KaKs_Calculator 2.0 was used to calculate non-synonymous substitution (Ka) and synonymous substitution (Ks) for tandemly duplicated genes [68].

### 4.6. Transcriptome Data Analysis

The raw data obtained from transcriptome sequencing were quality analyzed using FastQC v0.11.5 software, and the low-quality sequences were filtered by removing the joints (adapter) in the sequence files, and appropriate base modifications were made to filter the low-quality sequences by trim-galore v0.6.7 software. The clean data were postback compared to the pepper genome using STAR. The reads were precisely counted using the featureCounts v2.0.3 software. Differential expression analysis was performed with edgeR. The spatiotemporal expression properties of gene family members in pepper fruits were investigated by transcriptome data from 70 samples (PRJNA694629) from seven fruit developmental periods of ten pepper varieties [69]. Potential functions of family genes were explored by analyzing transcriptome data from four fruit developmental stages (PRJNA485468) of two pepper varieties with significant differences in fruit size [34]. The original pepper transcriptome sequencing data of fruits with different spiciness were derived from PRJNA779212 [35]. And an RNA-seq analysis was conducted on *Capsicum* (CM334) subjected to heat, cold, salt, and osmotic stresses at six different time points. A total of 78 RNA samples were used to construct the RNA-seq library, with three biological replicates at each time point for each abiotic stress and simulated control (PRJNA525913) [36].

Sample differential expression analysis data were performed using count values, and gene transcript expression heatmaps were performed using log2 (TMM + 1) values. The structural equation model was constructed using the plspm package (Partial Least Squares Structural Equation Modeling). All transcriptome data graphs were plotted by the R package.

### 4.7. PPI (Protein–Protein Interaction)

Protein interactions information of *Capsicum* transporters was analyzed by STRING database (https://string-db.org/, accessed on 7 October 2023) [70]. Graphical visualization was performed by Cytoscape v3.9.1 software [71]. 

### 4.8. Quantitative Real-Time PCR and PLS-SEM Analysis

*Capsicum* seedlings (CM334) were grown to the six-true-leaf stage in a light incubator. 50 mL of 400 mM mannitol solution was applied to simulate osmotic stress for 24 h, and water was applied to the corresponding control group. Total RNA was extracted from the leaves of pepper for reverse transcription, as described in the kit instructions (TaKaRa, Dalian, China). Primer design was performed by Primer Premier 6.0 software (primer sequences were listed in Supplementary Table S5), and real-time PCR amplification was carried out by an ABI7500 fluorescent quantitative PCR instrument (Agilent, Santa Clara, CA, USA). The constitutive actin gene (GenBank accession No. ACU27905) was used as the internal control and standard gene. PCR amplification conditions were as follows: 95 °C for 30 s, 40 cycles of 95 °C for 10 s, and 55 °C for 20 s, and 72 °C for 20 s. The relative expression of the gene was calculated by the 2^−ΔΔCt^ method. Graphs were plotted by GraphPad Prism v8 software. The values in the image are the averages of three biological replicates. 

Transcriptome data (PRJNA525913) from stress-treated pepper seedlings (CM334) were used for PLS-SEM (Partial Least Squares Structural Equation Modeling) analysis. The R package (plspm) was used for data analysis [72]. Each role of the family as a whole was abstracted as a latent variable, and the transcriptional strength of individual family members was treated as an observed variable.

## 5. Conclusions

In the present study, a comprehensive analysis of pepper sugar transporter genes was conducted to systematically identify and classify 50 *MST*, 3 *SUT*, and 29 *SWEET* genes in pepper at the genome-wide level. The expression patterns of pepper sugar transporter genes were analyzed by transcriptomic data from different fruit development periods. *Capsicum* sugar transporter genes had obvious spatiotemporal specific expression characteristics and complex transcriptional and translational regulation phenomena. The expression preference of sugar transporter genes among different varieties provides a potential application value for the improvement in pepper germplasm using genetic resources. The potential adversity-responsive functions of pepper sugar transporter genes were further analyzed by transcriptomic data of leaves under stress treatments. Structural equation modeling analysis revealed that the *MST*, *SUT*, and *SWEET* families jointly participated in the pepper adversity stress response by coordinating the expression of different members. The mode of action between the sugar transporters of pepper was maintained stable under high-temperature stress compared with low-temperature, osmotic stress, and salt stress. Our study may contribute to the functional mining of *Capsicum* sugar transporter genes and provide valuable clues for a deeper understanding of the molecular mechanisms of sugar transporter genes in pepper.

## Data Availability

The original contributions presented in the study are publicly available. The raw data for transcriptome sequencing of ten pepper varieties (Figure 3) for seven fruit developmental periods can be found here National Center for Biotechnology Information (NCBI) SRA database under accession number PRJNA694629 (Supplementary Table S6). The raw data for four developmental periods of two pepper varieties (Figure 4) with differing fruit types were obtained from NCBI under accession number PRJNA485468. The original pepper transcriptome sequencing data of fruits with different spiciness (Figure 5) and leaves (Figure 6) after stress treatment were derived from PRJNA779212 and PRJNA525913.

## Supplementary Materials

The following supporting information can be downloaded at: https://www.mdpi.com/article/10.3390/plants13131825/s1, Supplementary Table S1: Gene rename information. Supplementary Table S2: Protein physicochemical properties of gene family members. Supplementary Table S3: Ka/Ks calculation. Supplementary Table S4: Mantel test analysis. Supplementary Table S5: qPCR_primer. Supplementary Table S6: Pepper variety. Supplementary Figure S1: Analysis of gene structure and motif composition. Supplementary Figure S2: Phylogenetic tree of proteins. Supplementary Figure S3: Analysis of the expression trend of sugar transport gene in *Capsicum* under abiotic stress. Supplementary Figure S4: Principal component analysis. Supplementary Figure S5: Volcano plot of the differentially expressed genes. Supplementary Figure S6: Gene set enrichment analysis of sugar transporters in pepper. Supplementary Figure S7: MM-GS correlation scatter plot. Supplementary Figure S8: Protein–Protein Interaction Networks (PPIs) of sugar transporters in pepper.
