# Supplementary material for: Characterization and Expression Analysis of Sugar Transporters through Partial Least Square Structural Equation Model (PLS-SEM) Revealed Their Role in Pepper (Capsicum annuum L.)
Source: Plants (Basel). 2024 Jul 3;13(13):1825. doi: 10.3390/plants13131825 (PMC11243835; doi:10.3390/plants13131825)
Supplement: Supplementary file 1 [file plants-13-01825-s001.zip › Supplementary figures.pdf]

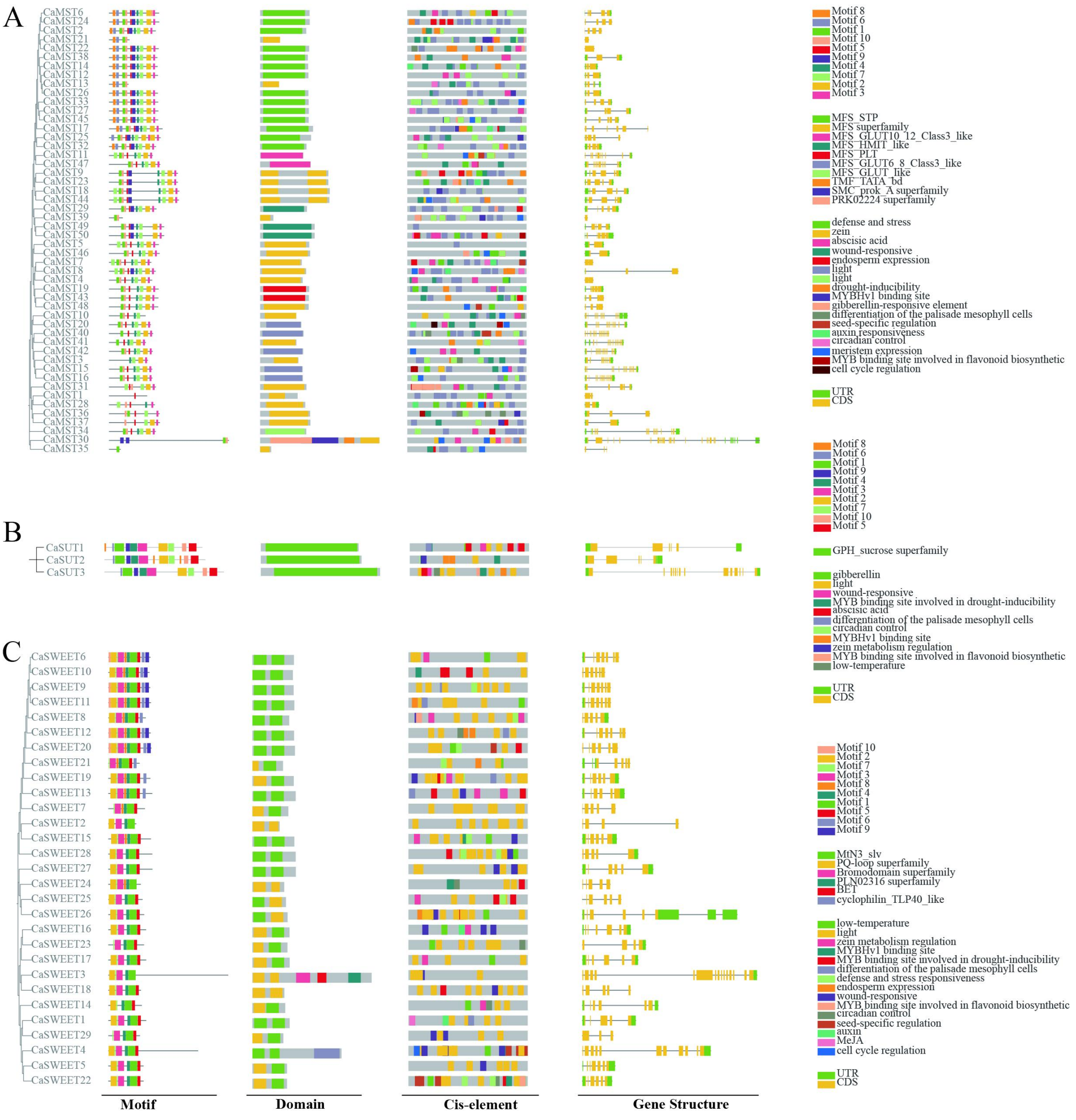

Supplmentary **Figure S1** Analysis of gene structure and motif composition. (**A**) MST gene family, (**B**) SUT gene family, (**B**) SWEET gene family.

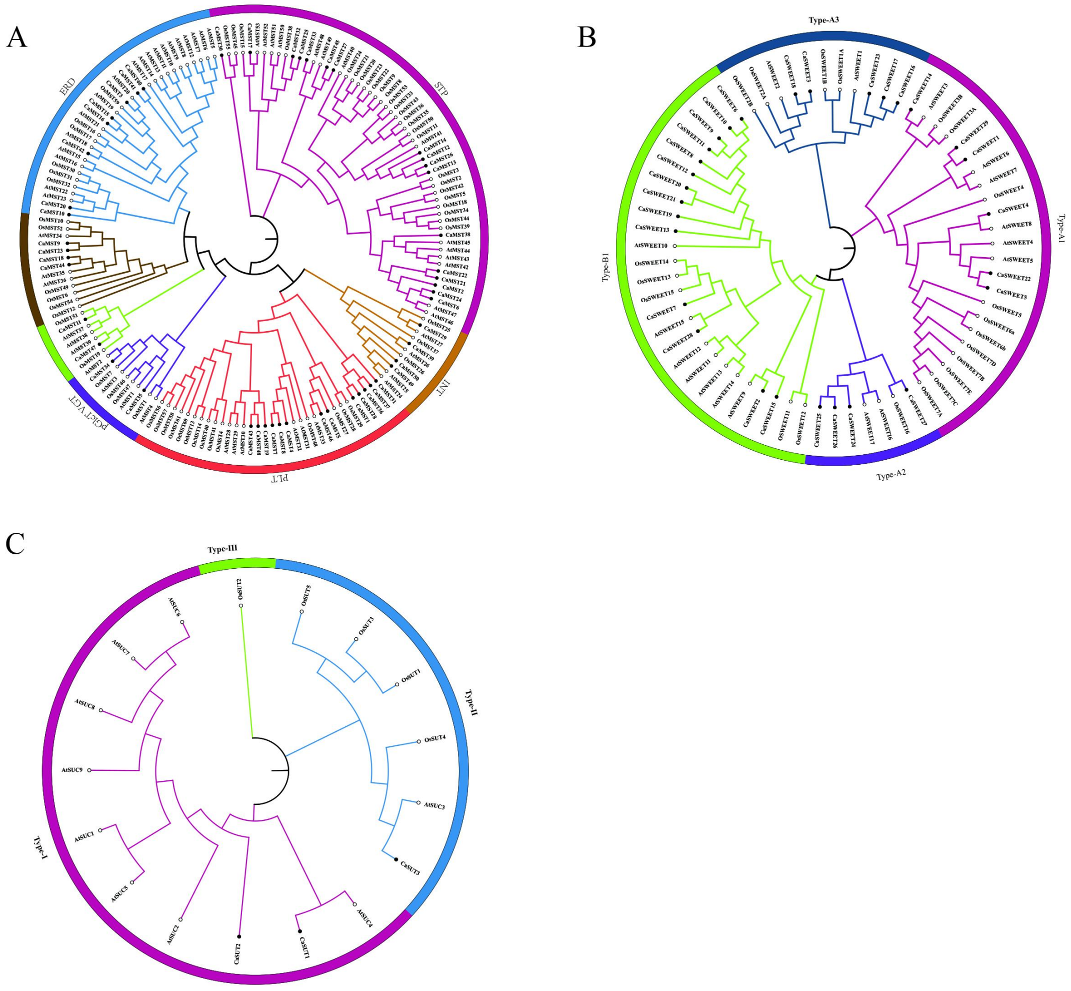

Supplementary **Figure S2** Phylogenetic tree of proteins. (**A**) MST gene family, (**B**) SWEET gene family, (**C**) MST gene family.

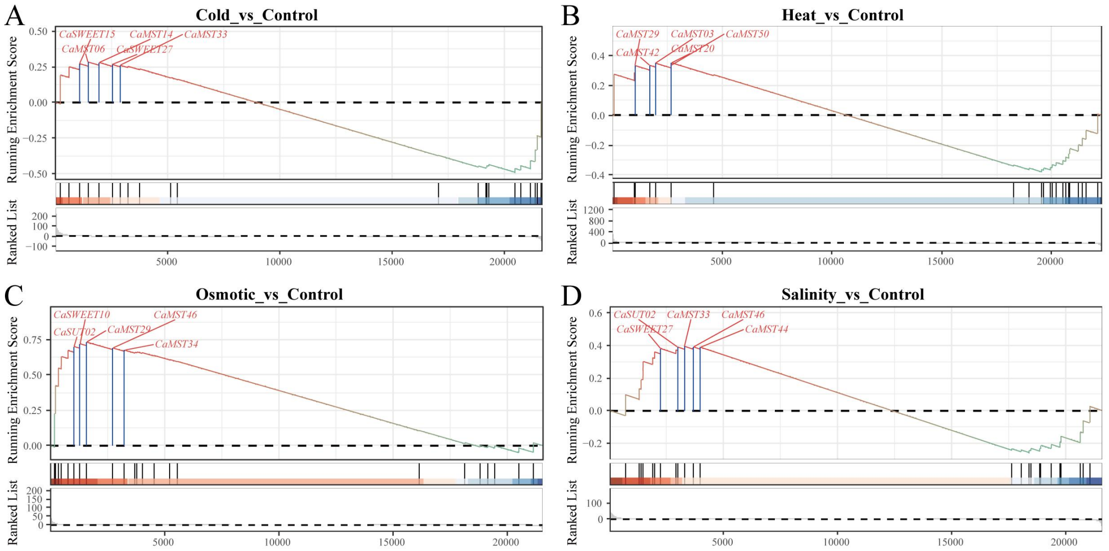

Supplementary **Figure S3** Analysis of the expression trend of sugar transport gene in *Capsicum* under abiotic stress. (**A**) Gene set enrichment analysis of sugar transporters under cold stress treatment. (**B**) Gene set enrichment analysis of sugar transporters under heat stress treatment. (**C**) Gene set enrichment analysis of sugar transporters under osmotic stress treatment. (**D**) Gene set enrichment analysis of sugar transporters under salinity stress treatment. All sugar transporters in pepper were regarded as a gene set. The red tags represent the top 5 genes in the queue. The horizontal axis is the sorted gene, the vertical axis is the corresponding enrichment score (ES), and the peak in the line graph is the ES of this gene set. ES starts from the first gene in the sequenced.

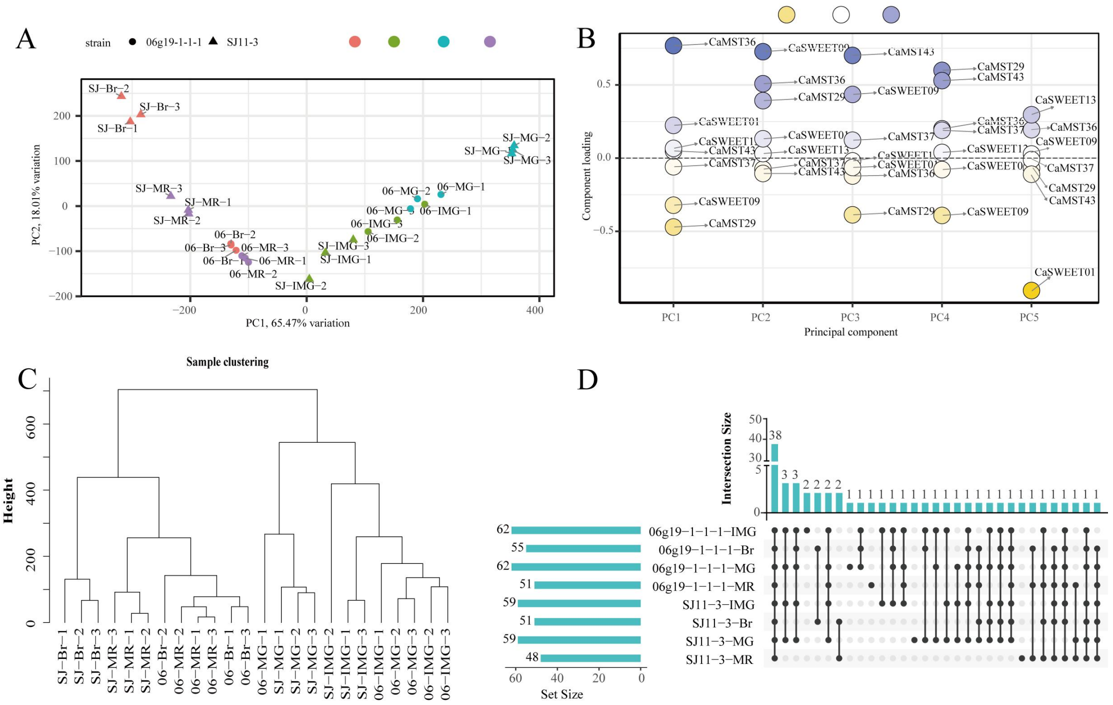

Supplementary **Figure S4** Principal component analysis. (**A**) Principal component analysis of 24 repeated samples of two pepper varieties, (**B**) Distribution of genes in the principal component dimension, (**C**) Hierarchical cluster analysis with sugar transporter genes between samples (**D**) Gene expression situation in samples.

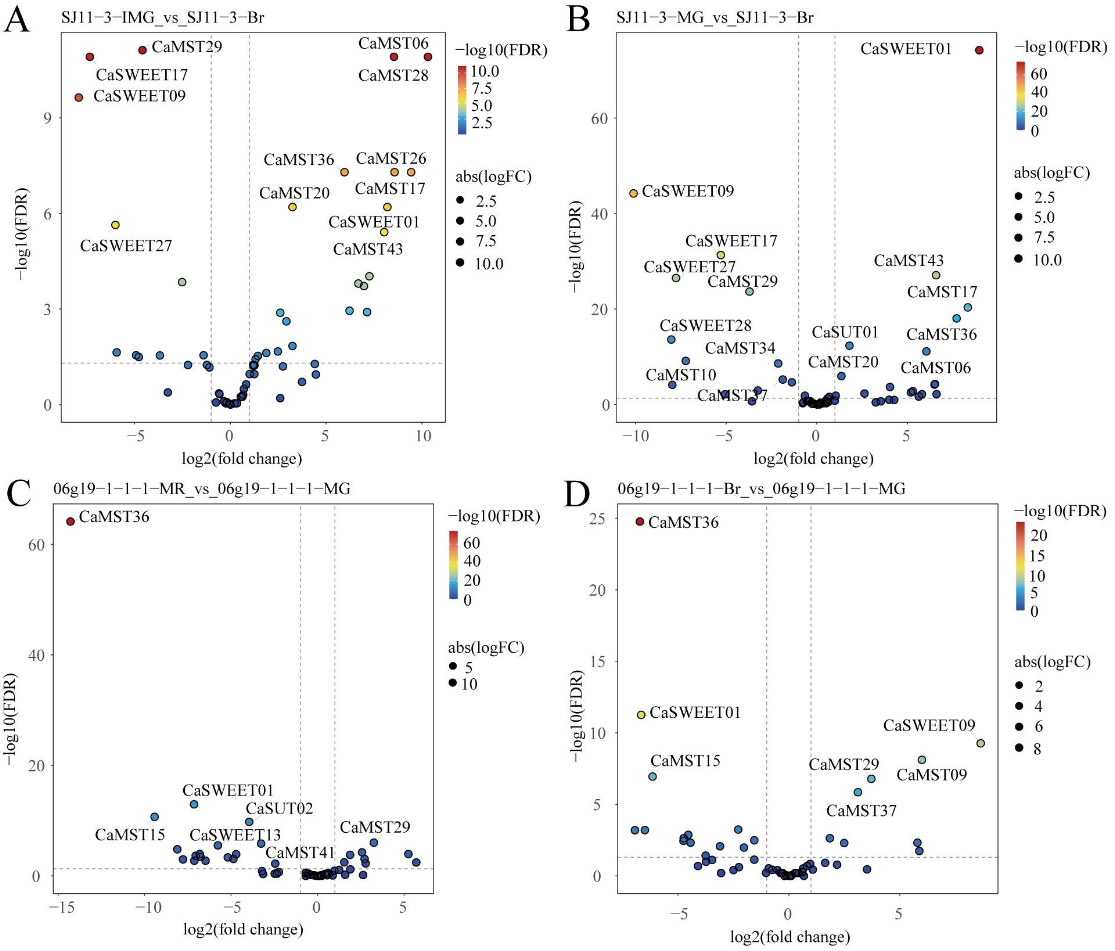

Supplementary **Figure S5** Volcano plot of the differentially expressed genes. (**A**) Differential expression of sugar transport genes at two fruit development stages: IMG and Br, in SJ11-3 pepper variety. (B) Differential expression of sugar transport genes at two fruit development stages, MG and Br, in SJ11-3 pepper variety. (**C**) Differential expression of sugar transport genes at MR and MG fruit development stages in the 06g19-1-1-1 pepper variety. (**D**) Differential expression of sugar transport genes at two fruit development stages, Br and MG, in the 06g19-1-1-1 pepper variety. The X coordinate was log2(fold change) and the Y coordinate was -log10(FDR). Each dot represented a gene.

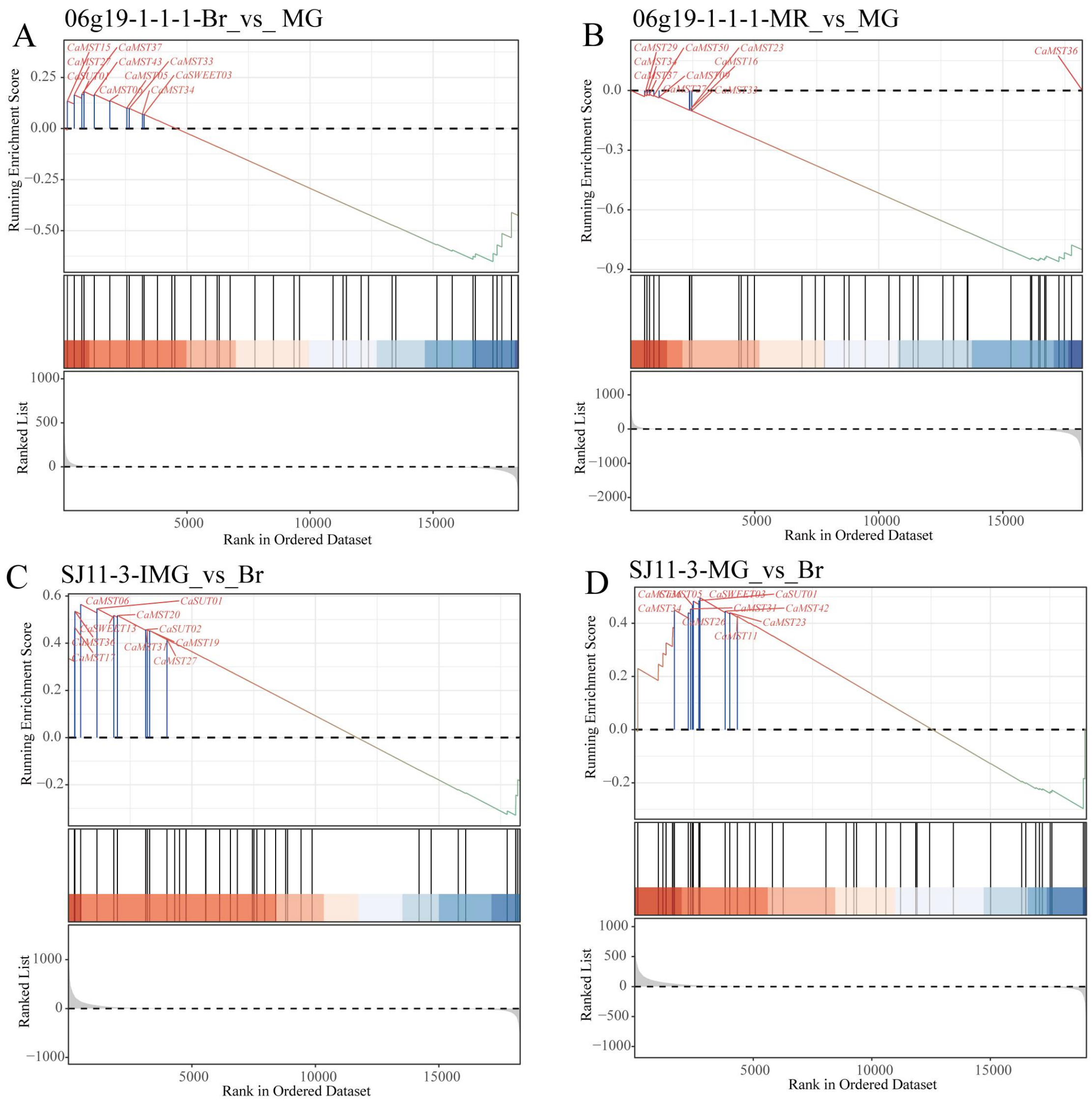

Supplementary **Figure S6** Gene set enrichment analysis of sugar transporters in pepper. (**A**) Gene set enrichment analysis of sugar transporters at two fruit development stages: Br and MG, in 06g19-1-1-1 pepper variety. (**B**) Gene set enrichment analysis of sugar transporters at two fruit development stages: MR and MG, in 06g19-1-1-1 pepper variety. (**C**) Gene set enrichment analysis of sugar transporters at two fruit development stages: IMG and Br, in SJ11-3 pepper variety. (**D**) Gene set enrichment analysis of sugar transporters at two fruit development stages: MG and Br, in SJ11-3 pepper variety. All sugar transporters in pepper were regarded as a gene set. The red tags represent the top 10 genes in the queue. The horizontal axis is the sorted gene, the vertical axis is the corresponding enrichment score (ES), and the peak in the line graph is the ES of this gene set. ES starts from the first gene in the sequenced.

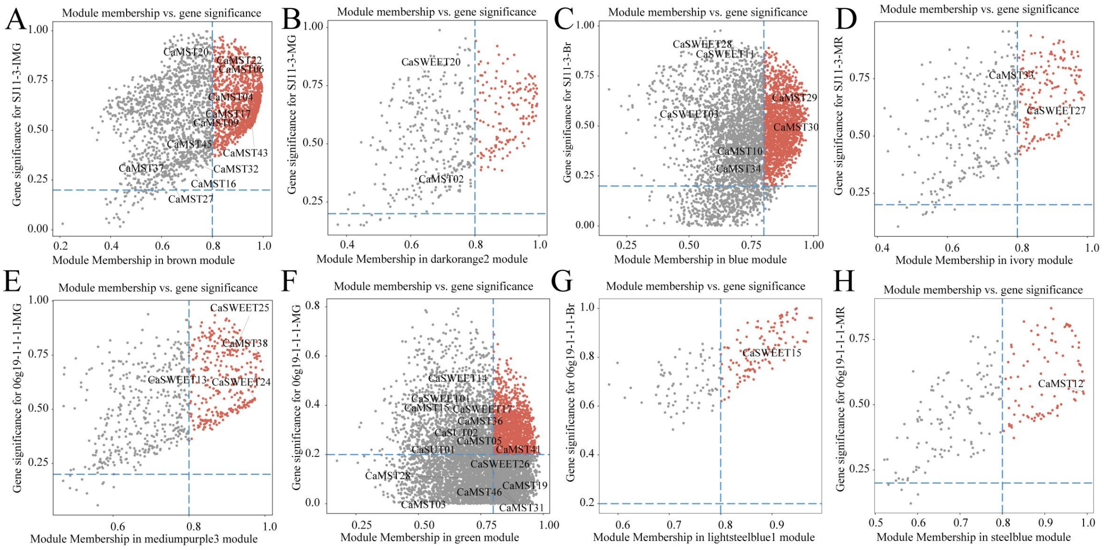

Supplementary **Figure S7** MM-GS correlation scatter plot. (**A**) Brown module of SJ11-3 at IMG period of fruit development. (**B**) Darkorange2 module of SJ11-3 at MG period of fruit development. (**C**) Blue module of SJ11-3 at Br period of fruit development. (**D**) Ivory module of SJ11-3 at MR period of fruit development. (**E**) Mediumpurple3 module of 06g19-1-1-1 at IMG period of fruit development. (**F**) Green module of 06g19-1-1-1 at MG period of fruit development. (**G**) Lightsteelblue1 module of 06g19-1-1-1 at Br period of fruit development. (**H**) Steelblue module of 06g19-1-1-1 at MR period of fruit development. The ordinate indicates the correlation between genes and traits: gene significance GS; the abscissa indicates the correlation between genes and modules: module membership. MM was defined as the correlation of the module eigengene and the gene expression profile. Gene Significance GS was defined as the absolute value of the correlation between the gene and the trait.

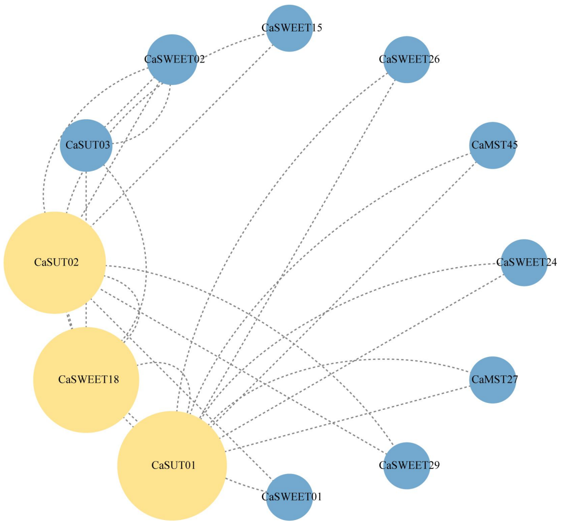

Supplementary **Figure S8** Protein-Protein Interaction Networks (PPI) of sugar transporters in pepper.
